# Normal variation of the gut microbiota affects hepatic cytochrome P450 activity in mice

**DOI:** 10.1002/prp2.893

**Published:** 2021-11-08

**Authors:** Masao Togao, Shinnosuke Tajima, Takashi Kurakawa, Gaku Wagai, Jun Otsuka, Shoichi Kado, Koji Kawakami

**Affiliations:** ^1^ Safety Research Department Yakult Central Institute Kunitachi‐shi Tokyo Japan; ^2^ Basic Research Department Yakult Central Institute Kunitachi‐shi Tokyo Japan

**Keywords:** 16S rRNA gene, cytochrome P450, drug metabolism, intestinal flora, normalization

## Abstract

Several studies revealed that substantial artificial changes in the gut microbiota resulted in modification of hepatic cytochrome P450 3a (Cyp3a) in mice. Consequently, we hypothesized that “normal” variation of the gut microbiota might also alter hepatic Cyp activity and lead to individual differences in drug metabolism. Therefore, this study investigated the effects of normal gut microbiota variation on hepatic Cyp activity under the same genetic and environmental conditions using ex‐germ‐free mice. Using the feces of three breeder BALB/c mice (Jcl, Slc, and Crj), germ‐free BALB/cYit mice were conventionalized (Yit‐Jcl, Yit‐Slc, and Yit‐Crj). The gut microbiota composition and hepatic Cyp activity of these donors and recipients were evaluated. 16S rRNA sequencing revealed clear differences of the gut microbiota among donors and among recipients. Cyp3a activity was significantly higher in Slc mice than in Jcl and Crj mice. Notably, among recipients, Cyp3a activity was significantly higher in Yit‐Slc and Yit‐Crj mice than in Yit‐Jcl mice. Cyp2b activity was significantly higher in Slc mice than in Jcl and Crj mice. Cyp2b activity was significantly higher in Yit‐Slc mice than in Yit‐Jcl mice. Additionally, in correlation analysis, some genera displayed significant positive or negative correlations with Cyp activity, particular the strong positive correlation between *Clostridium sensu stricto 1* with Cyp3a activity. In conclusion, this study demonstrated that normal variation of the gut microbiota affected hepatic Cyp3a and Cyp2b activity, which might result in individual differences of drug metabolism.

## Graphical abstract

Using ex‐GF mice (Yit‐Jcl, Yit‐Slc, and Yit‐Crj) conventionalized with feces from mice obtained from three breeders (Jcl, Slc, and Crj), we revealed that normal variation of the gut microbiota could affect hepatic Cyp activity, and several bacterial groups might modulate Cyp activity.

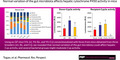

**Abbreviations**
CYP: cytochrome P450 (humans)
Cyp: cytochrome P450 (mice)
GF: germ‐free
LDA: linear discriminant analysis
LEfSe: linear discriminant analysis effect size
OTU: operational taxonomic unit
SPF: specific pathogen‐free

## 1 INTRODUCTION

The gut microbiota plays a key role in maintaining host health through many aspects.^1^, ^2^, ^3^ Drug metabolism is also regarded to be influenced by the gut microbiota.^4^, ^5^ Ample studies demonstrated the link between the gut microbiota and the hepatic activity of cytochrome P450 (Cyp),^6^, ^7^, ^8^ a major drug‐metabolizing enzyme. In particular, previous papers comparing germ‐free (GF) and specific pathogen‐free (SPF) mice reported that in the absence of the gut microbiota, the expression and activity of hepatic Cyp3a were clearly depressed,^8^, ^9^ leading to decreased Cyp3a drug metabolism in vivo and increased drug concentrations in plasma and tissues.^10^ CYP3A is the dominant isoform of CYP, and it plays a significant role in the metabolism of approximately 50% of marketed drugs,^11^ therefore, it is possible that the gut microbiota affects the metabolism of many drugs.

Regarding the influence of “variation” of the gut microbiota on hepatic Cyp, previous research concluded that gastrectomy in mice increased the hepatic expression of Cyp3a and the other major isoform Cyp2b by increasing pH in the gastrointestinal tract and modifying the gut microbiota.^12^ Other studies demonstrated that antibiotic‐treated mice, which exhibited disruption of the gut microbiota, displayed lower hepatic Cyp3a and Cyp2b expression.^6^, ^7^ These findings indicated that substantial artificial alteration of the gut microbiota resulted in the modification of hepatic Cyp3a and Cyp2b expression and activity. Likely because of difficulties of excluding genetic and environmental factors that can directly affect Cyp activity, no reports have demonstrated the impact of “normal” variation such as interindividual variation of the gut microbiota on hepatic Cyp activity.

Wide individual differences of the gut microbiota^13^ and CYP activity^14^ have been described, even among healthy people. Concerning CYP activity, genetic polymorphism is considered a major cause of individual difference, but the contribution of other unknown factors must also be considered.^15^ These findings led us to hypothesize that normal variation of the gut microbiota could contribute to CYP activity and result in individual differences of drug metabolism.

Thus, this study investigated the effects of normal gut microbiota variation on hepatic Cyp activity under the same genetic and environmental condition by conventionalizing GF mice using different gut microbiota samples obtained from three mouse breeders.

## 2 MATERIALS AND METHODS

### 2.1 Chemicals

Isopropanol, ethanol, sucrose, glycerol, Tris, EDTA‐2K, potassium hydrogen phosphate, and potassium dihydrogen phosphate were purchased from Fujifilm Wako Pure Chemical Co.. Meanwhile, 1 M Tris‐HCl (pH 9.0), 0.5 M EDTA (pH 8.0), 10% SDS, TE buffer, TE‐saturated phenol, phenol/chloroform/isoamyl alcohol (25:24:1), and 3 M sodium acetate (pH 5.2) were purchased from Nippon Gene Co., Ltd..

### 2.2 Animals

As feces donors, male 8‐week‐old SPF BALB/cAJcl, BALB/cCrSlc, and BALB/cAnNCrlCrlj mice (Jcl, Slc, and Crj groups, respectively; *n* = 6/group) were purchased from CLEA Japan, Inc., Japan SLC, Inc., and Charles River Laboratories Japan, Inc., respectively. As feces recipients, male 4‐week‐old GF BALB/cYit mice (*n* = 18) were obtained from the breeding colony of Yakult Central Institute for conventionalization using Jcl (Yit‐Jcl), Slc (Yit‐Slc), and Crj (Yit‐Crj) feces (*n* = 6/group). In addition, male GF BALB/cYit (Yit‐Cont) mice were used as an untreated group (*n* = 6).

All mice were housed in cages in flexible film isolators. The breeding room was controlled under a 12‐h/12‐h light/dark cycle. Room temperature and humidity were maintained at 23 ± 3°C and 50% ±20%, respectively. Radiation‐sterilized chow (FR‐2 50 kGy, Funabashi Farm Co., Ltd.) and sterilized drinking water were available ad libitum. The sterility of GF mice was confirmed via microscopic and culture examination.

All experiments using animals were conducted under the supervision of the Institutional Animal Care and Use Committee of Yakult Central Institute and approved by the director of the Yakult Central Institute (approval number: 19‐0070). All animals were cared for and used under a program accredited by AAALAC International.

### 2.3 Treatments

Mice from each group were housed in separate isolators and permitted to acclimatize for 2 weeks. At 10 weeks of age, donor mice were removed from the isolators after the collection of feces. Then, 4‐week‐old recipient mice were moved from the GF isolator to the corresponding donor isolator. Nine pieces of donor feces were suspended in 3 ml of saline solution and 0.15 ml of fecal homogenates were orally administered to each recipient mouse. The recipient mice were then raised until reaching 10 weeks old. Additionally, Yit‐Cont mice were raised until 10 weeks of age in separate isolators.

At 10 weeks of age, all mice were removed from their isolators after feces collection. Then, the animals were weighed and exsanguinated from the posterior vena cava and abdominal aorta under isoflurane anesthesia, and the liver of each animal was harvested at the same time on different days. After weighing, livers and feces were frozen in liquid nitrogen and then stored at −80°C until use.

### 2.4 Gut microbiota analysis

DNA was extracted from feces using glass beads and phenol as described previously.^16^ The total counts of bacteria in feces were measured by quantitative PCR using a previously described method^17^ with slight modifications. Briefly, each reaction mixture (20 μl) was composed of 10 μl of 2× TB Green^®^ Premix Ex Taq™ II (Takara Bio), 0.04 μl each of 100 µM forward primer (UniF: 5′‐GTGSTGCAYGGTCGTCA‐3′) and 100 µM reverse primer (UniR: 5′‐ACGTCRTCCMCNCCTTCCTC‐3′),^18^ 0.4 μl of 50× ROX Reference Dye II, 4.52 μL of nuclease‐free water (QIAGEN N.V.), and 5 μl of DNA. PCR was performed using the AB7500 system (Applied Biosystems) using the following conditions: 94°C for 5 min followed by 40 cycles of 94°C for 20 s, 60°C for 20 s, and 72°C for 50 s. The fluorescent products were detected at the last step of each cycle. The total number of bacteria per gram of feces was calculated by substituting the Ct value obtained from the amplification curve of each sample into the standard curve, which was generated with 10‐fold serial dilutions of DNA extracted from *Faecalibacterium prausnitzii* YIT 12316^T^. The cell counts of the standard bacterial strain were determined microscopically using 4′,6‐diamidino‐2‐phenylindole staining as described previously.^19^

### 2.5 16S rRNA sequencing

The V4 region of the bacterial 16S rRNA gene was amplified and sequenced using the primers 515F (5′‐GTGCCAGCMGCCGCGGTAA‐3′) and 806R (5′‐GGACTACHVGGGTWTCTAAT‐3′) according to a previously described method^20^ with small modifications. Briefly, bar‐coded amplicons were generated using TB Green Premix Ex Taq II (Takara Bio) with 10 ng of template fecal DNA. The PCR program was as follows: 95°C for 30 s followed by 30 cycles of 95°C for 5 s, 50°C for 30 s, and 72°C for 40 s. The reaction was stopped immediately before DNA amplification reached a plateau. The amplicons were purified using an AMPure XP Kit (Beckman Coulter) and their concentrations were quantified using a Quant‐iT™ PicoGreen™ dsDNA Assay (Invitrogen). The amplicons were pooled in equimolar amounts and then sequenced on a MiSeq system (Illumina) with a MiSeq Reagent Kit v2 500 cycle (Illumina).

The obtained sequence data were processed using QIIME2^21^ (version 2020. 8) with silva (version 138) as a reference database to obtain the operational taxonomic unit (OTU) table (File S1) and the relative abundances of bacteria at the phylum and genus levels. Differences in occupancy among groups were determined via linear discriminant analysis (LDA) effect size (LEfSe) analysis^22^ with the condition of LDA score >4.0 using the Galaxy application (http://huttenhower.sph.harvard.edu/galaxy/). Principal coordinate analysis was used to visualize the difference of composition based on the unweighted and weighted UniFrac distances. The count of each constituent bacterium was calculated by multiplying the total number of bacteria by the occupancy. In addition, as alpha diversity indices, observed OTUs, Faith's phylogenetic diversity (Faith's PD), and the Shannon index were calculated using 20 000 reads per sample.

### 2.6 Cyp activities

Hepatic microsomes were prepared using previously described methods.^10^ The total protein concentration was measured using a Pierce™ BCA Protein Assay Kit (Thermo Fisher Scientific). Cyp3a, Cyp2b, and Cyp2c activities in hepatic microsomes were measured using the P450‐Glo™ Assay (Promega) according to the manufacturer's instructions and a previous report.^10^ In detail, a luminogenic substrate, i.e., luciferin IPA for Cyp3a, luciferin 2B6, for Cyp2b, and luciferin H for Cyp2c, and hepatic microsomes in 0.2 M potassium phosphate buffer (pH 7.4) were preincubated for 10 min at 37°C. After adding NADPH regeneration systems (Promega), the reaction mixtures were incubated for 10, 20, or 30 min to measure Cyp3a, Cyp2b, or Cyp2c activity, respectively, at 37°C. Then, luciferin detection reagent was added and stabilized for 20 min at room temperature. Luminescence was measured using a LUMIstar OPTIMA (BMG LABTECH). As positive controls, Supersomes of CYP3A4, CYP2B6, and CYP2C9 (Corning) for luciferin IPA, luciferin 2B6, and luciferin H, respectively, were used. Similarly, Supersomes‐CYP minus (Corning) served as a negative control.

### 2.7 Gene expression levels in the liver

mRNA was extracted from small pieces of liver tissue using the ReliaPrep™ Miniprep System (Promega). First‐strand cDNA was generated from approximately 1000 ng of total RNA using Rever Tra Ace qPCR RT Master Mix (Toyobo). cDNA was examined by real‐time PCR using PowerUp™ SYBR™ Green Master Mix (Applied Biosystems) and the AB7500 system. As target genes, in addition to Cyp3a11, Cyp2b10, and Cyp2c29, which are the major mouse isoforms of Cyp3a, Cyp2b, and Cyp2c, respectively,^23^ pregnane X receptor (Pxr), a major transcriptional regulator of Cyp3a11 that is involved in the regulation of Cyp2b and Cyp2c,^24^ organic anion transport polypeptide C (Oatpc), a downstream gene of Pxr,^25^ and constitutive androstane receptor (Car), a major transcriptional regulator of Cyp2b10,^24^ were evaluated. The sequence of each primer is presented in Table S1. The delta‐delta Ct method was used to calculate the relative levels of mRNA. β‐actin was used for normalization.

### 2.8 Statistical analysis

The Steel–Dwass method was used to compare the total counts of bacteria and alpha diversity indices among donors and among recipients. PERMANOVA was used to evaluate the difference of composition based on the unweighted and weighted UniFrac distances. The Bonferroni‐corrected *t*‐test method was used to compare hepatic Cyp activity and gene expression among donors and among recipients. Spearman's rank correlation analysis was used to evaluate the association between Cyp activity and the counts of constituent bacteria at the phylum and genus levels by pooling donor and recipient samples (the analysis was performed using the bacterial groups in which more than half of the samples were detected). A *p* value less than .05 was considered statistically significant. Two Yit‐Crj individuals died after conventionalization, and they were therefore excluded from the analysis. All statistical analyses were performed using Bell Curve for Excel (Social Survey Research Information Co., Ltd.).

## 3 RESULTS

### 3.1 Gut microbiota

**FIGURE 1:**
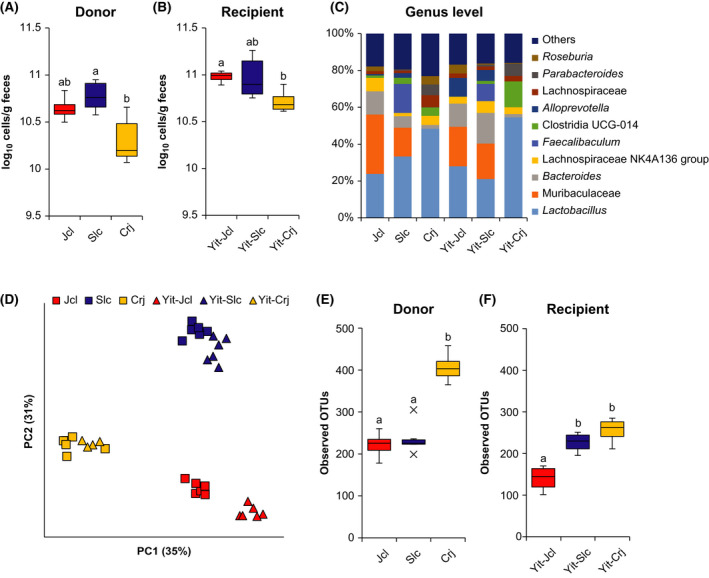
Characteristics of the gut microbiome in donor and recipient mice. (A) Total counts of bacteria in feces from donor mice. (B) Total counts of bacteria in feces from recipient mice. (C) Fecal bacterial composition of donor and recipient mice at the genus level. (D) Principal coordinates analysis plot based on the unweighted UniFrac distances of fecal bacteria from donor and recipient mice. (E) Observed operational taxonomic units (OTUs) in feces from donor mice. (F) Observed OTUs in feces from recipient mice. The Steel–Dwass method was used to compare the total counts of bacteria and alpha diversity indices among donors and among recipients. Different letters (a and b) indicate significant differences among donors and among recipients

The total bacterial counts in donor and recipient mouse feces are presented in Figure 1A,B. Crj and Yit‐Crj mice had the lowest total bacterial counts among donor and recipient mice, respectively. In detail, the total bacterial count was significantly higher in Slc mice than in Crj mice (*p* = .028). Similarly, Yit‐Jcl mice had a significantly higher bacterial count than Yit‐Crj mice (*p* = .049).

The bacterial composition at the phylum (Figure S1) and genus levels (Figure 1C) in each group is presented as cumulative bar charts. At the phylum level, Bacteroidota and Firmicutes comprised more than 90% of the bacteria in all groups, suggesting that they are the major components of the microbiota. Crj and Yit‐Crj mice had a lower proportion of Bacteroidota and a higher proportion of Firmicutes than the other groups. At the genus level, the bacterial composition varied greatly among the groups. In Jcl, Slc, Yit‐Jcl, and Yit‐Slc mice, Muribaculaceae genus unidentified and *Lactobacillus* were dominant, whereas *Lactobacillus* was the most dominant bacterial genus in Crj and Yit‐Crj mice, with Muribaculaceae genus unidentified comprising less than 0.1% of the bacterial population. Additionally, LEfSe revealed the characteristics at other taxonomic levels in each mouse group (Figure S2).

To visualize the variation of gut microbiota in donors and recipients, the results of principal coordinate analysis based on unweighted and weighted UniFrac distances are presented in Figure 1D and Figure S3. Based on unweighted UniFrac distances, there were significant differences in the gut microbiota composition among donors and recipients (*p* < .01). Conversely, the gut microbiota composition was similar between donors and their corresponding recipients. Likewise, based on weighted UniFrac distances, the gut microbiota of Yit‐Crj mice was significantly different from those of Yit‐Jcl and Yit‐Slc mice (*p* < .01). As indicators of alpha diversity, the observed OTUs (Figure 1E,F), Faith's PD, and Shannon's index (Figure S4) were measured. The observed OTUs were significantly higher in Crj mice than in Jcl and Slc mice (*p* = .011). Additionally, the observed OTUs were significantly higher in Yit‐Slc and Yit‐Crj mice than in Yit‐Jcl mice (*p* = .011 and *p* = .028, respectively). Faith's PD was significantly larger in Crj mice than in Jcl and Slc mice (*p* = .011 and *p* = .018, respectively), and the index was significantly larger in Slc mice than in Jcl mice (*p* = .011). Among recipient mice, Faith's PD was significantly larger in Yit‐Slc and Yit‐Crj mice than in Yit‐Jcl mice (*p* = .011 and *p* = .028, respectively). Concerning Shannon's index, no significant difference was found among donor mice, whereas the value was significantly higher in Yit‐Slc mice than in Yit‐Crj mice (*p* = .028).

### 3.2 Hepatic Cyp activities

**FIGURE 2:**
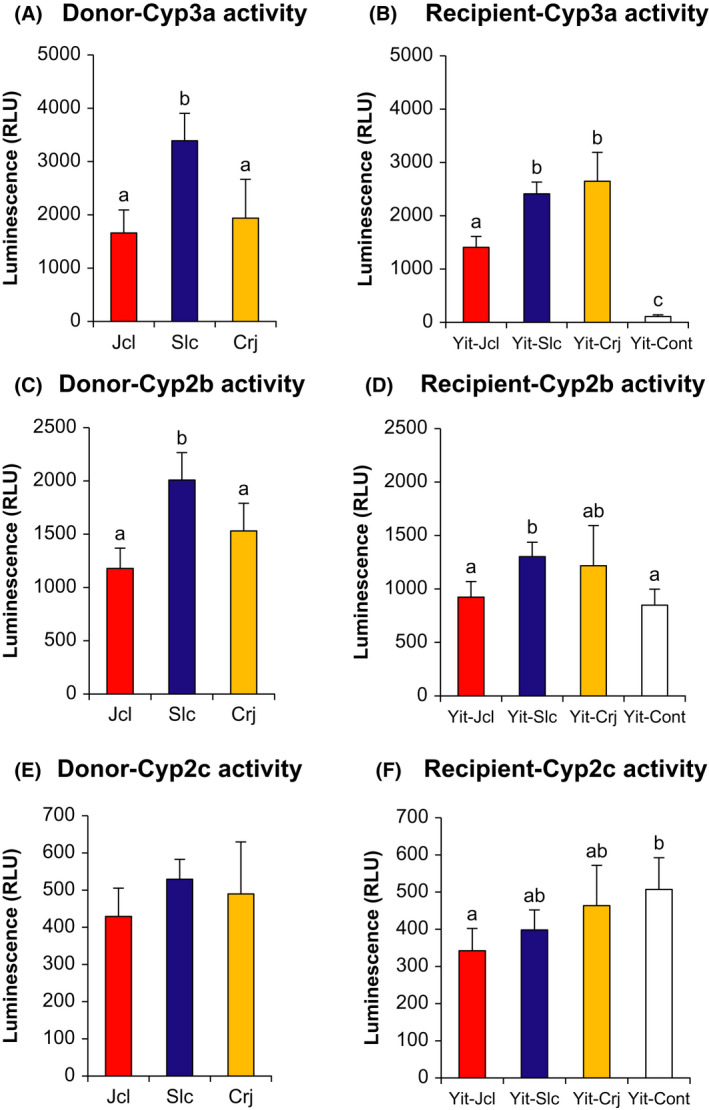
Cytochrome P450 (Cyp) activities in liver microsomes prepared from donor and recipient mice using a chemiluminescence assay. (A) donor Cyp3a, (B) recipient Cyp3a, (C) donor Cyp2b, (D) recipient Cyp2b, (E) donor Cyp2c, (F) recipient Cyp2c. The Bonferroni‐corrected *t*‐test method was used to compare hepatic Cyp activity among donors and among recipients. Different letters (a, b, and c) indicate significant differences among donors and among recipients. Data are expressed as the mean ± standard deviation

Hepatic Cyp activities in donors and recipients are presented in Figure 2. In donors, Cyp3a activity was significantly higher in Slc mice than in Jcl and Crj mice (*p* < .001 and *p* = .002, respectively). Among recipients, Cyp3a activity was significantly higher in all recipient groups than in the Yit‐Cont group (*p* < .001). Furthermore, Cyp3a activity was significantly higher in Yit‐Slc and Yit‐Crj mice than in Yit‐Jcl mice (*p* < .001). Cyp2b activity was significantly higher in Slc mice than in Jcl (*p* < .001) and Crj mice (*p* = .010). Cyp2b activity was significantly higher in Yit‐Slc mice than in Yit‐Cont mice (*p* = .006). Moreover, Cyp2b activity was significantly higher in Yit‐Slc mice than in Yit‐Jcl mice (*p* = .027). There was no significant difference in Cyp2c activity among the donor groups. By contrast, Cyp2c activity was significantly lower in Yit‐Jcl mice than in Yit‐Cont mice (*p* = .009).

### 3.3 Hepatic Cyp and related gene expression

**FIGURE 3:**
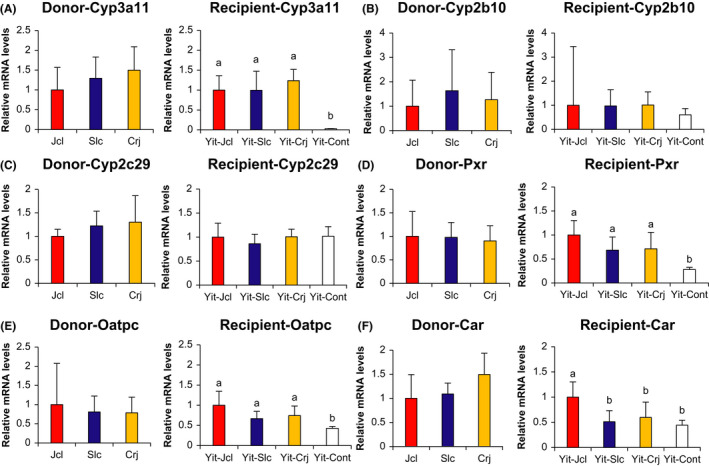
Hepatic expression of cytochrome P450 (Cyp) and related genes in donor and recipient mice using the delta‐delta Ct method. (A) Cyp3a11, (B) Cyp2b10, (C) Cyp2c29, (D) pregnane X receptor (Pxr), (E) organic anion transport polypeptide C (Oatpc), (F) Constitutive androstane receptor (Car). The Bonferroni‐corrected *t*‐test method was used to compare hepatic gene expression among donors and among recipients. Different letters (a and b) indicate significant differences among recipients. Data are expressed as the mean ± standard deviation

The hepatic expression of Cyp and related genes in donors and recipients is presented in Figure 3. Cyp3a11 expression was similar among the donor groups, whereas its expression was significantly higher in all recipient groups than in the Yit‐Cont group (*p* < .001). However, Cyp3a11 expression did not differ among the recipient groups. The expression of both Cyp2b10 and Cyp2c29 was equivalent among the donor and among recipient groups.

Regarding nuclear factor expression, Pxr, Oatpc, and Car expression did not differ among the donor groups. Conversely, Pxr and Oatpc expression was significantly higher in all recipient groups than in the Yit‐Cont group (*p* < .001 and *p* < .05, respectively). Car expression was significantly higher in Yit‐Jcl mice than in Yit‐Slc and Yit‐Cont mice (*p* = .008 and *p* = .001, respectively).

### 3.4 Association between Cyp activity and gut microbiota

**FIGURE 4:**
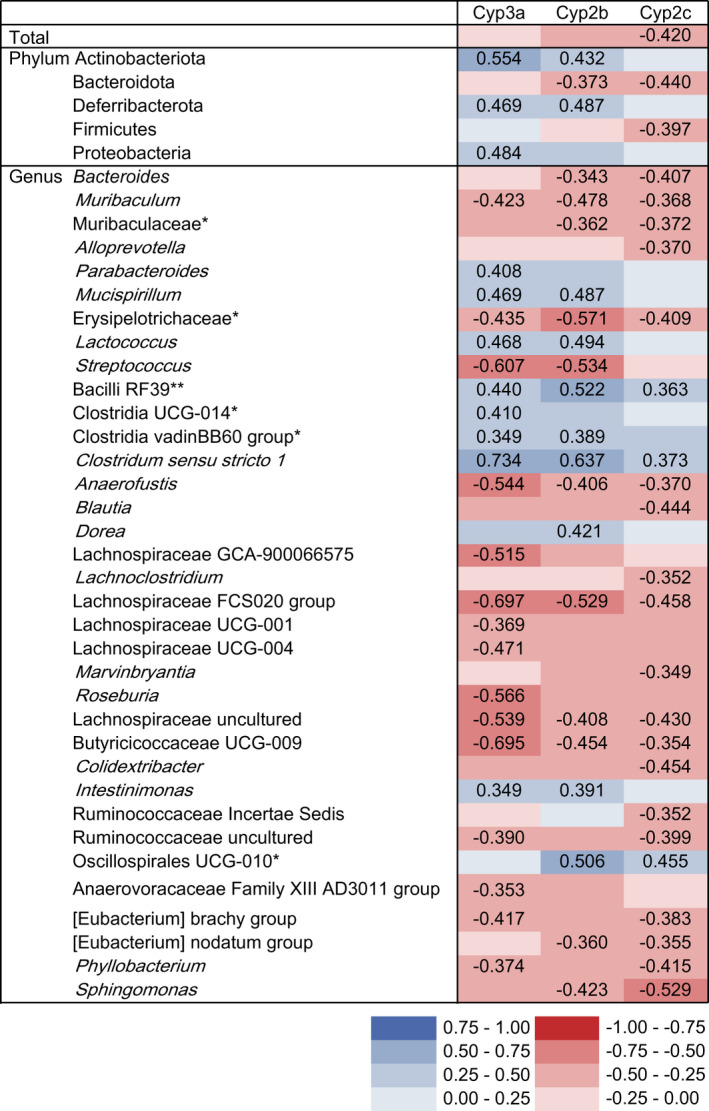
Spearman's rank correlation heat map between bacterial counts and cytochrome P450 (Cyp) activity. Only phyla and genera that were significantly correlated with any of the Cyp isoforms are listed. rs values are presented for significant correlations. *Means genus unidentified. **Means family and genus unidentified

Correlation analysis was performed to examine the relationship between Cyp activity and gut bacterial counts (Figure 4). Positive correlations with Cyp activity were observed for several phyla and genera. Specifically, Actinobacteriota, Deferribacterota, and Proteobacteria displayed significant positive correlations with Cyp3a activity, and at the genus level, *Parabacteroides*, *Mucispirillum*, *Lactococcus*, Bacilli RF39, Clostridia UCG‐014, Clostridia vadinBB60 group, *Clostridium sensu stricto 1*, and *Intestinimonas* exhibited significant positive correlations with Cyp3a activity. In particular, *Clostridium sensu stricto 1* displayed a strong positive correlation with Cyp3a activity (rs = 0.734, *p* < .001).

Concerning Cyp2b activity, Actinobacteriota and Deferribacterota exhibited significant positive correlations at the phylum level, and *Mucispirillum*, *Lactococcus*, Bacilli RF39, Clostridia vadinBB60 group, *Clostridium sensu stricto 1*, *Dorea*, *Intestinimonas*, and Oscillospirales UCG‐010 had significant positive correlations at the genus level.

No phylum displayed a positive correlation with Cyp2c activity, whereas the genera Bacilli RF39, *Clostridium sensu stricto 1*, and Oscillospirales UCG‐010 exhibited significant positive correlations.

Meanwhile, the total bacterial count had a significant negative correlation with Cyp2c activity. At the phylum level, Bacteroidota displayed significant negative correlations with Cyp2b and Cyp2c activity, and Firmicutes exhibited a significant negative correlation with Cyp2c activity. Furthermore, several genera displayed significant negative correlations with Cyp activity.

## 4 DISCUSSION

Using ex‐GF mice conventionalized with feces from mice obtained from three breeders, we revealed that normal variation of the gut microbiota could affect hepatic Cyp activity, and correlation analysis illustrated that several bacterial groups might modulate Cyp activity.

Regarding the gut microbiota, principal coordinate analysis based on unweighted UniFrac distances revealed clear differences in the gut microbiota composition among donors and among recipients. The result in donors was consistent with previous findings revealing breeder‐based differences in the gut microbiota composition in C57BL/6 mice.^26^, ^27^ The present study also identified similar microbiota compositions between donors and their corresponding recipients, indicating that the recipient generally inherited the microbiota of the donor. These results illustrated that recipients maintained the normal variation of the gut microbiota under the same genetic and environmental backgrounds. Therefore, the differences among recipients, if any, are considered attributable to the difference in the gut microbiota composition in the present study.

Regarding donor Cyp activity, Cyp3a and Cyp2b activities in Slc were higher than in the other donors and no significant difference in Cyp2c activity was observed. The same result was also obtained in our preliminary study in which commercially available mice livers from each breeder were used to measure the hepatic Cyp activity (data not shown). Therefore, the difference is not by chance but owing to specific differences among these breeder mice.

Regarding Cyp3a activity in recipients, all recipient mouse groups had higher activity than the Yit‐Cont group. This result is consistent with those of previous conventionalization studies.^8^ It must be emphasized that Yit‐Slc and Yit‐Crj mice had higher Cyp3a activity than Yit‐Jcl mice. These results indicated that the induction efficiency of hepatic Cyp3a activity following conventionalization depends on the colonizing bacteria. Additionally, as Yit‐Crj mice had lower total bacterial counts in the gut microbiota and high hepatic Cyp3a activity, it is suggested that the bacterial composition has a stronger impact on Cyp3a activity than total bacterial counts. Taken together, these results suggested that the gut microbiota greatly enhanced hepatic Cyp3a activity, and even within the normal range, variation of the gut microbiota can lead to differences in Cyp3a activity.

Cyp2b activity was higher in Yit‐Slc mice than in Yit‐Jcl and Yit‐Cont mice. However, Yit‐Jcl and Yit‐Crj mice did not have significantly different Cyp2b activity than Yit‐Cont mice. Concerning the effects of the microbiota on Cyp2b activity, the results obtained to date are controversial. Kuno et al. reported higher Cyp2b activity in SPF C57BL/6NCrSlc mice obtained from Japan SLC, Inc. than in GF mice.^6^ Conversely, Li et al. found that Cyp2b activity was lower in conventional C57BL/6J mice obtained from Jackson Laboratory than in GF mice.^28^ In this regard, the breeder in the former study^6^ was the same as that used in the present study. It follows that bacteria that enhance Cyp2b activity might be present in the gut microbiota of Slc mice. Further study is required to determine the precise effects of the gut microbiota on hepatic Cyp2b activity.

Cyp2c activity was lower in Yit‐Jcl mice than in Yit‐Cont mice. An earlier study reported that gut microbiomes enhanced Cyp2c activity,^29^ however, we previously reported lower Cyp2c activity in SPF mice than in GF mice in a study using BALB/cAJcl mice^10^ which is the same strain with Jcl in this study. Thus, bacteria that attenuate Cyp2c activity could be present in the gut microbiota of Jcl mice. Taken together, these results suggested that even under normal conditions, the composition of the gut microbiota could attenuate the activity of certain Cyp isoforms including Cyp2c.

Regarding Cyp gene expression, Cyp3a11 expression was higher in all recipient groups than in the Yit‐Cont group, consistent with previous conventionalization studies.^8^, ^29^, ^30^ These findings suggested the existence of gut microbiomes enhanced Cyp3a activity by modulating gene expression. Contrarily, there were no differences in Cyp2b10 and Cyp2c29 among all recipient groups compared with the Yit‐Cont group. The results of Cyp2b10 is consistent with previous conventionalized mice studies.^8^, ^30^ Furthermore, there were no differences among donors or recipients regarding Cyp3a11, Cyp2b10, and Cyp2c29 expression, and thus, the results for Cyp gene expression were not consistent with those for Cyp activity. In this regard, discrepancies between mRNA and activity of Cyp are common and have also been reported in previous reports using conventional and GF mice.^28^, ^31^ These differences could be attributable to, for example, post‐transcriptional regulation. Some reports indicated that CYPs including CYP3A, CYP2B, and CYP2C isoforms are significantly controlled by microRNA.^32^, ^33^ These effects might explain the difference between gene expression and Cyp activity.

The expression of the nuclear receptor Pxr and its downstream gene Oatpc was higher in all recipient groups than in the Yit‐Cont group. This was also recorded in previous reports, which identified significant upregulation of Pxr in conventionalized mice.^8^ Taken together, it is possible that the gut microbiota modulates Cyp gene expression via Pxr, which influences Cyp activity.

The expression of the nuclear receptor Car was higher in Yit‐Jcl mice than in Yit‐Slc and Yit‐Cont mice. However, Cyp2b10 gene expression did not differ among the groups, whereas Cyp2b activity was lower in Yit‐Jcl mice than in Yit‐Slc mice but similar to that in Yit‐Cont mice. Thus, the present study could not support the contribution of Car to the effects of the gut microbiota on Cyp activity.

Correlation analysis revealed that several phyla and genera had positive correlations with Cyp activity, suggesting that differences of the bacterial composition in the gut microbiota enhanced Cyp activity. It has been reported that secondary bile acids produced mainly by *Lachnoclostridium* spp. such as *Clostridium scindens* and *Clostridium hylemonae* can modify hepatic Cyp activity.^7^, ^9^ In this study, no clear correlation between *Lachnoclostridium* counts and Cyp activity was observed. Contrarily, *Clostridium sensu stricto 1* had positive correlations with Cyp3a, Cyp2b, and Cyp2c activities. In particular, a strong correlation of *Clostridium sensu stricto 1* was observed with Cyp3a activity. The previous study reported that *Clostridium butyricum*, which belongs to *Clostridium sensu stricto 1*, enhanced the transcription of MicroRNA‐200c in Caco‐2 BBe cells,^34^ and evidence suggests that MicroRNA‐200c affects human hepatic CYP3A4 activity.^33^ In addition, *Clostridium butyricum* produces high levels of butyrate,^35^ which is suggested to induce certain Cyp isoforms.^30^ Taken together, it is possible that *Clostridium sensu stricto 1* spp. can enhance Cyp activity. Further studies are needed to evaluate the precise effects of individual bacteria on the modulation of Cyp activity and elucidate the responsible mechanisms.

At the same time, several phyla and genera exhibited negative correlations with Cyp activity. Although the mechanisms were not clarified in this study, it is possible that the bacteria in these phyla and genera are involved in the attenuation of Cyp activity, including that of Cyp2c, which was attenuated by the gut microbiota in the present study. Therefore, the gut microbiota may be involved in both the enhancement and attenuation of Cyp activity.

There are several limitations in this study. First, we only obtained data about Cyp activity; thus, whether normal variation of the gut microbiota affects in vivo drug metabolism or the clearance of clinically relevant drugs is uncertain. Furthermore, there could be a species difference in effects of microbiota on Cyp activity. Hence, further study is required to address these problems.

In conclusion, this study demonstrated that normal variation of the gut microbiota affected hepatic Cyp activity, including Cyp3a activity, in mice. Additionally, it was estimated that several bacterial groups can modulate Cyp activity. Our results suggest that normal variation of the gut microbiota could contribute to hepatic CYP activity and explain the individual differences in drug metabolism in humans.

## DISCLOSURE

The authors declare no conflicts of interest associated with this manuscript.

## AUTHOR CONTRIBUTIONS

Participated in research design: Togao, Tajima, Wagai, Otsuka, and Kawakami. Conducted experiments: Togao, Tajima, Kurakawa, and Kawakami. Performed data analysis: Togao, Kurakawa, and Kawakami. Wrote or contributed to the writing of the manuscript: Togao, Tajima, Kurakawa, Wagai, Otsuka, Kado, and Kawakami.

## ETHICS APPROVAL STATEMENT

All experiments using animals were conducted under the supervision of the Institutional Animal Care and Use Committee of Yakult Central Institute and approved by the director of the Yakult Central Institute (approval number: 19‐0070). All animals were cared for and used under a program accredited by AAALAC International.

## Supporting information

Fig S1

Fig S2

Fig S3

Fig S4

Supplementary Material

Supplementary Material

## Data Availability

Research data are not shared.
