# Supplementary material for: Normal variation of the gut microbiota affects hepatic cytochrome P450 activity in mice
Source: Pharmacol Res Perspect. 2021 Nov 8;9(6):e00893. doi: 10.1002/prp2.893 (PMC8573722; doi:10.1002/prp2.893)
Supplement: Supplementary file 5 — Supplementary Material [file PRP2-9-e00893-s004.docx]

**Supplemental Table**

# Supplemental Table 1. Sequence of RT-qPCR primers

| Primer | Forward (5′–3′) | Reverse (5′–3′) | Reference |
| --- | --- | --- | --- |
| Cyp3a11 | ACAAACAAGCAGGGATGGAC | GGTAGAGGAGCACCAAGCTG | (Li et al., 2017) |
| Cyp2b10 | AAGGAGAAGTCCAACCAGCA | CTCTGCAACATGGGGGTACT | (Li et al., 2017) |
| Cyp2c29 | AGCCTACTGTCATATTGCACGGGT | CATGCCCAAATTTCGCAGGGTCAT | (Suzuki et al., 2020) |
| Pxr | CCCATCAACGTAGAGGAGGA | TCTGAAAAACCCCTTGCATC | (Zhang et al., 2014) |
| Oatpc | AGGACACCAAGACTGCTGGTG | CCCAAGCTTTCTGGCATTTG | (Li et al., 2009) |
| Car | CTCAAGGAAAGCAGGGTCAG | AGTTCCTCGGCCCATATTCT | (Zhang et al., 2014) |
| β-actin | GGCCAACCGTGAAAAGATGA | CAGCCTGGATGGCTACGTACA | (Li et al., 2017) |

# Cyp, cytochrome P450; Pxr, pregnane X receptor; Oatpc, organic anion transport polypeptide C; Car, constitutive androstane receptor

Li CY, Lee S, Cade S, Kuo LJ, Schultz IR, Bhatt DK, Prasad B, Bammler TK, and Cui JY (2017) Novel interactions between gut microbiome and host drug-processing genes modify the hepatic metabolism of the environmental chemicals polybrominated diphenyl ethers. *Drug Metab Dispos* 45: 1197-1214.

Li Y, Ross-Viola JS, Shay NF, Moore DD, and Ricketts M-L (2009) Human CYP3A4 and murine Cyp3A11 are regulated by equol and genistein via the pregnane X receptor in a species-specific manner. *J Nutr* 139: 898-904.

Suzuki S, Nishijima C, Sato Y, Umegaki K, Murata M, and Chiba T (2020) Coleus forskohlii extract attenuated the beneficial effect of diet-treatment on nash in mouse model. *J Nutr Sci Vitaminol (Tokyo)* 66: 191-199.

Zhang YW, Bao MH, Hu L, Qu Q, and Zhou HH (2014) Dose-response of oridonin on hepatic cytochromes P450 mRNA expression and activities in mice. *J Ethnopharmacol* 155: 714-720.

**Supplemental figure legends**

Supplemental Figure 1. Bacterial compositions in feces from donor and recipient mice at the phylum level.

Supplemental Figure 2. Linear discriminant analysis (LDA) of bacterial taxa. Taxonomic groups with LDA scores exceeding 4.0 are presented. Taxonomic levels are represented as p (phylum), c (class), o (order), f (family), and g (genus).

Supplemental Figure 3. Principal coordinate analysis plot based on the weighted UniFrac distance of bacteria in feces from donor and recipient mice.

Supplemental Figure 4. Faith’s phylogenetic diversity (PD) and Shannon’s index of feces from donor and recipient mice. (A) Faith’s PD in donor mice. (B) Faith’s PD in recipient mice. (C) Shannon’s index of feces from donor mice. (D) Shannon’s index of feces from recipient mice. Different letters (a, b, and c) indicate significant differences among donors and among recipients.
